# Case report: Spontaneous lens absorption after the implantation of an implantable collamer lens

**DOI:** 10.3389/fmed.2023.1202691

**Published:** 2023-08-03

**Authors:** Xiaojing Liu, Qiulin Zeng, Ting Lu, Min Wang, Yong Sun, Jingfa Zhang

**Affiliations:** ^1^Department of Ophthalmology, Jiading Branch of the Shanghai General Hospital, Shanghai Jiao Tong University School of Medicine, Shanghai, China; ^2^Department of Ophthalmology, Shanghai Xinshijie Eye Hospital, Shanghai, China; ^3^Department of Ophthalmology, Shanghai General Hospital, Shanghai Jiao Tong University School of Medicine, Shanghai, China

**Keywords:** spontaneous absorption of lens, ICL, cataract, anterior capsular fibrotic proliferation, anterior subcapsular opacification

## Abstract

**Background:**

Spontaneous lens absorption is rare and usually occurs in eyes with certain syndromes, hyper-mature cataracts, and ocular trauma. The application of an implantable collamer lens (ICL) is widely performed in patients with high myopia for refractive correction. This study reports a case of spontaneous lens absorption after ICL implantation.

**Case summary:**

A 23-year-old man was referred with complaints of poor vision in his left eye. The patient had undergone binocular ICL implantation for refractive correction of high myopia 1.5 years prior. Approximately 10 months later, he experienced a sudden loss of vision and pain in his left eye, which resolved spontaneously the next day without any treatment. Since then, the visual acuity in his left eye gradually decreased. At presentation, slit-lamp examination revealed an ICL in the posterior chamber of both eyes, with anterior capsular fibrotic proliferation and posterior capsular opacity, and the residual lens cortex sandwiched between the anterior fibrotic membrane and opacified posterior lens capsule in his left eye. The number of corneal endothelial cells in his left eye was 1,337, which was lower than before ICL implantation (2,902). The patient then underwent ICL extraction, anterior capsular capsulotomy, residual cortex aspiration, posterior capsular polishing, and intraocular lens implantation.

**Conclusions:**

Spontaneous lens absorption may occur in patients with ICL implantation. Patients should undergo routine follow-ups after ICL implantation.

## Highlights

- Spontaneous lens absorption is rarely reported in patients with ICL implantation.
- The lytic lens cortex may cause chronic uveitis and phacolytic glaucoma.
- Patients with sudden or gradual vision loss should be cautioned and advised to undergo routine follow-up after ICL implantation.

## Introduction

An implantable collamer lens (ICL) is considered one of the safest and most advanced technologies for the correction of myopia. Unlike keratorefractive surgery, ICLs are placed in the eye through minimally invasive surgery without damaging the cornea. Patients receive clearer uncorrected vision after correction, and ICL surgery is becoming a new trend in myopia correction technology. ICL implantation benefits patients with high myopia who cannot undergo keratorefractive surgery due to insufficient corneal thickness. The ICL is placed in the posterior chamber of the eye and fixed in the ciliary sulcus, achieving the goal of long-term correction of ametropia.

The main type of ICL is a one-piece intraocular lens with a central port. The central port allows sufficient aqueous flow from the posterior chamber to the anterior chamber to avoid pupillary block and maintain normal IOP (1). Parameters such as ICL size and vault are associated with the safety of ICL surgery. The degree of variation in the vault is related to the interaction of the lens implant with the anatomy and physiology of the posterior chamber.

Spontaneous lens absorption is rare and usually occurs in eyes with certain syndromes, hyper-mature cataracts, and ocular trauma (2). Lens absorption after ICL implantation has not yet been reported. In this study, we describe a case of spontaneous transparent lens absorption after ICL implantation.

### Timeline

**Figure 1:**
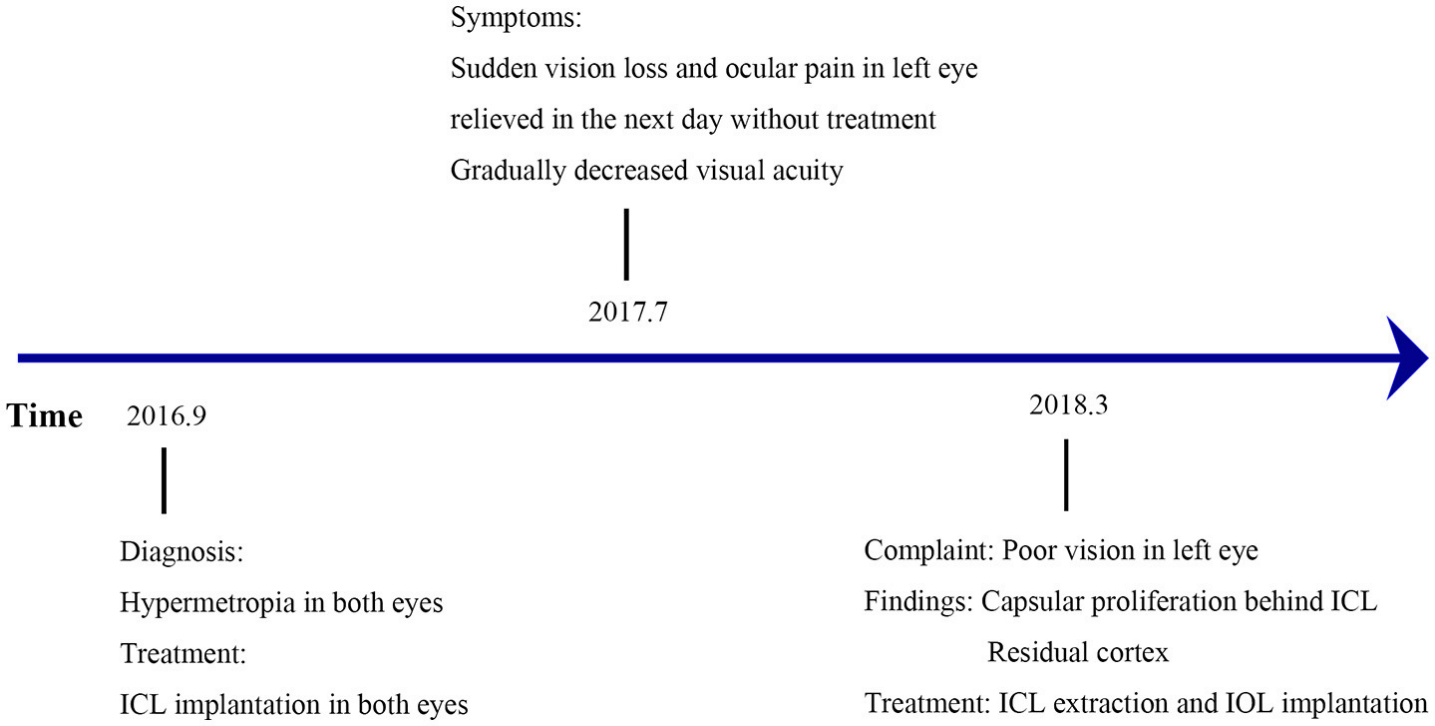
Timeline of the patient before and after ICL implantation. ICL, implantable collamer lens; IOL, intraocular lens.

The present case showed the absorption of the transparent lens after ICL implantation was found in March, 2018, which was 1.5 years after ICL implantation. The timeline for this patient was shown in Figure 1.

## Case presentation

### Chief complaints

A 23-year-old man complained of gradual loss of vision in his left eye for more than 6 months.

### History of the present ophthalmic disease

The patient experienced a sudden loss of vision and ocular pain in his left eye in July 2017. The symptoms had relieved spontaneously the following day without any treatment, and his visual acuity had gradually decreased since then. Carteolol hydrochloride eye drops had been administered to both eyes twice a day since February 2018 to control the increased intraocular pressure.

### Past medical history

The patient had no history of trauma or systemic disease. No family history of ocular disease was found. The patient had undergone ICL implantation (V4C) in both eyes for myopia correction in another hospital 1.5 years ago (September 2016). A slight opacity was noted at follow-up, which was localized at the inferior center of the anterior capsule 1 month after ICL implantation.

### Physical examination

**Figure 2:**
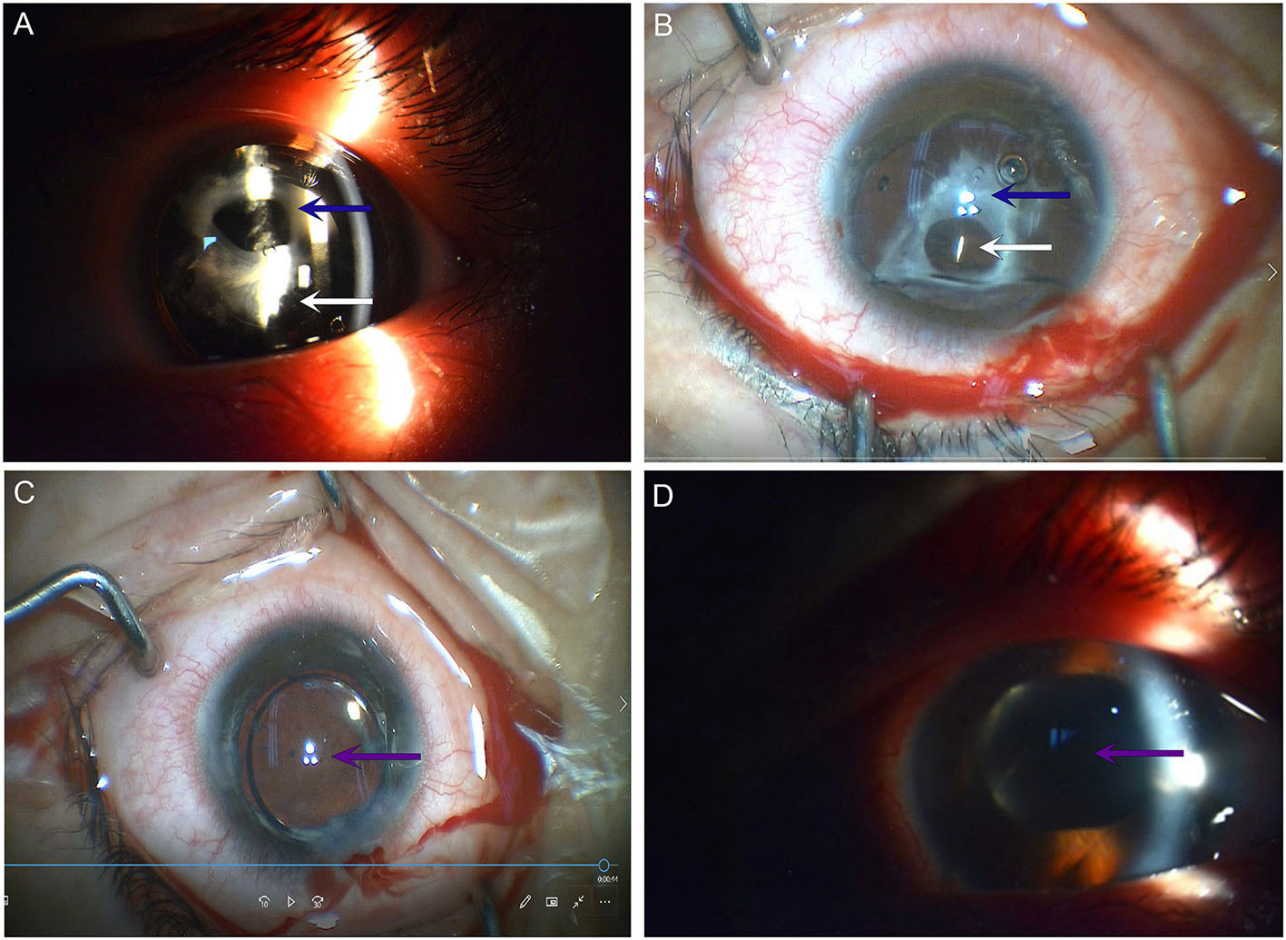
Ophthalmic examination before and after ICL extraction. **(A)** Photograph taken under slit-lamp examination before ICL extraction. **(B)** The photograph was taken under a surgical microscope before ICL extraction. **(C)** The photograph was taken under a surgical microscope after ICL extraction. **(D)** Photograph taken under slit-lamp examination 1 day after ICL extraction. A blue arrow indicates the white anterior capsular proliferation ring; a white arrow indicates the ICL; and a purple arrow indicates the IOL. ICL, implantable collamer lens; IOL, intraocular lens.

Visual acuity was finger count (FC)/20 cm in the left eye and 0.6 (logMAR) in the right eye. The intraocular pressure was 24.3 mmHg (right eye) and 22.3 mmHg (left eye). A slit-lamp examination showed an orthophoric ICL in both eyes, with no abnormal findings in the right eye. In the left eye, however, the anterior chamber depth was normal, the pupil was nearly round with a diameter of 4 mm, and it was slow to react to light stimulation. Temporal segmental atrophy was observed in the iris. In addition, the thick, whitish materials behind the ICL were detected in the left eye (Figure 2). The residual lens cortex was noticed, which was sandwiched between the anterior white capsular proliferation ring band and the posterior opacified lens capsule. There were no signs of active uveitis. The fundus of the left eye was not clearly visible by the slit-lamp or any other imaging system. The number of corneal endothelial cells in his left eye was 1,337, which was lower than the number before ICL implantation (2,902) and in the contralateral eye (2,627).

## Results and follow-up

The findings confirmed the diagnosis of spontaneous lens absorption in the left eye. The patient underwent the following surgery in the left eye: ICL extraction, separation of the anterior-posterior capsular synechiae, capsulotomy of the anterior capsular proliferation ring, aspiration of the residual lens cortex, and polishing of the posterior lens capsule, in addition to the implantation of an intraocular lens (Johnson & Johnson Tecnis sensor ar40e) into the ciliary sulcus. The visual acuity was 0.8 (logMAR) 1 day after surgery.

## Discussion

Spontaneous lens absorption is rarely reported and usually occurs in eyes with certain syndromes, hyper-mature cataracts, and ocular trauma. In this study, for the first time, we reported a case of spontaneous lens absorption after ICL implantation.

ICL implantation is a safe and effective procedure for myopia correction, and implantable phakic contact lenses (IPCL) are rapidly increasing. This is a cost-effective method to substitute ICL. However, there are some differences between ICL and IPCL. ICL is a phakic intraocular lens designed to be implanted in the ciliary sulcus. It is made of Collamer^®^, a biocompatible material, and has four soft haptics for atraumatic contact with the sulcus. The ICL has five holes to ensure adequate aqueous flow between both sides of the intraocular lens (IOL). On the other hand, IPCL is a phakic plate-shaped intraocular lens designed for posterior chamber implantation. It is made of a reinforced hybrid hydrophilic acrylic material with haptics designed to ensure gentle contact with the sulcus. It has 11 holes designed to maintain an adequate aqueous flow between both sides of the IOL (3).

Complications after ICL or IPCL implantation are not rare, the most common being cataract formation (4). Anterior subcapsular opacification is the most prevalent type of cataract (43%), followed by posterior subcapsular opacification. The incidence of anterior subcapsular opacification varies, with ICL being 40% and IPCL being 100% 1 year after the implantation. The high incidence of anterior subcapsular opacification after IPCL surgery may be related to direct contact between the implant and the crystalline lens or to altered aqueous humor circulation with subsequent lens malnutrition, but it is independent of implant material (5). Other complications, such as pupillary block glaucoma and toxic anterior segment syndrome causing pupillary block, are unusual (5, 6). However, there is currently no literature on the relationship between implanted crystal materials and spontaneous lens absorption after ICL or IPCL implantation.

The exact causal reasons and mechanisms for lens absorption remain unclear and are likely to vary depending on the cause. As is well-known, ICL implantation is becoming a routine clinical procedure widely used for the correction of refractive errors with fewer complications. An injury to the lens capsule may be responsible for spontaneous lens absorption, as in traumatic cases (7, 8). In our case, an iatrogenic capsular rupture could be largely excluded because there were no complications reported by this patient for at least 10 months after ICL implantation, and the anterior chamber depth was normal at his presentation, with the only positive sign being the anterior capsular rupture. However, other causes should be considered. In one case of intraocular foreign body (9), it was suggested that the lens cortex was emulsified and spontaneously prolapsed from the absorbed lens. Osmotic forces due to chemical changes on both sides of the lens capsule have also been postulated as playing a role (2, 7, 8). Siderosis was also implicated as a cause of lens absorption (9). Similarly, the rubella virus has been isolated in cases of spontaneous lens absorption from either clear lens material of infants with congenital rubella syndrome or cataractous lens material, even at 35 months of age (2, 10, 11).

In our study, although the exact reason was not known, the slight opacity recorded to be localized in the inferior center of the anterior capsule 1 month after ICL implantation may indicate a small tear/break of the anterior capsule of the lens that occurred for unknown reasons, possibly during or after the ICL surgery. The white circular proliferation ring band of the anterior capsule was clearly evidenced by the absorption of the lens cortex 1.5 years after ICL implantation. The patient's sudden loss of vision and pain may have been caused by uveitic glaucoma due to lens material leakage through the rupture. In addition to those symptoms, evidence of secondary glaucoma in this patient was iris segmental atrophy. The pain and the possible corneal edema caused by the ocular pain may relieve while intraocular pressure decreased on the second day.

Following capsular rupture, lens protein that has broken down and liquefied may leak through an intact capsule. The leaked lens cortical material could act as a potent antigenic stimulus, producing an inflammatory response in the eye and causing chronic uveitis or phacolytic glaucoma. Although some liquefied proteins have lost their antigenicity and do not cause uveitis (12), the induced macrophage response may result in secondary glaucoma (13). Moreover, if the liquefied proteins are released at a slower rate, they may not cause phacolytic glaucoma. The transient symptoms of ocular pain and gradual loss of vision described by our patient may be caused by the above reasons since the sign of segmental atrophy of the iris was observed in the left eye at the first visit.

## Conclusions

Spontaneous lens absorption may occur in patients with ICLs. Patients should undergo routine follow-ups after this type of implantation.

## Data availability statement

The original contributions presented in the study are included in the article/Supplementary material, further inquiries can be directed to the corresponding authors.

## Ethics statement

Written informed consent was obtained from the individual(s) for the publication of any potentially identifiable images or data included in this article.

## Author contributions

XL, QZ, TL, and MW reviewed the literature, contributed to the drafting, and discussion of the manuscript. XL, YS, and JZ analyzed and interpreted the imaging findings. YS contributed to the acquisition of funding. YS and JZ are guarantors of this work, who had full access to all the data in this study and take responsibility for their integrity and accuracy. All authors approved the final version to be submitted.

## Supplementary material

Supplementary Figure 1 The corneal endothelial cell count and ultrasound biomicroscopic examination of the patient. **(A)** The corneal endothelial cell count of the left eye 2 days before ICL implantation. **(B)** The corneal endothelial cell count of both eyes 1.5 years after ICL implantation. **(C)** Ultrasound biomicroscopic examination of the left eye 1 week before ICL extraction.
